# Routine versus selective cardiac magnetic resonance in non-ischemic heart failure – OUTSMART-HF: study protocol for a randomized controlled trial (IMAGE-HF (heart failure) project 1-B)

**DOI:** 10.1186/1745-6215-14-332

**Published:** 2013-10-12

**Authors:** Ian Paterson, George A Wells, Justin A Ezekowitz, James A White, Matthias G Friedrich, Lisa M Mielniczuk, Eileen O’Meara, Benjamin Chow, Rob A deKemp, Ran Klein, Carole Dennie, Alexander Dick, Doug Coyle, Girish Dwivedi, Miroslaw Rajda, Graham A Wright, Mika Laine, Helena Hanninen, Eric Larose, Kim A Connelly, Howard Leong-Poi, Andrew G Howarth, Ross A Davies, Lloyd Duchesne, Seppo Yla-Herttuala, Antti Saraste, Paul Farand, Linda Garrard, Jean-Claude Tardif, Malcolm Arnold, Juhani Knuuti, Rob Beanlands, Kwan L Chan

**Affiliations:** 1 Mazankowski Alberta Heart Institute, University of Alberta, Edmonton, AB, Canada; 2 Division of Cardiology, (including Cardiac Imaging, The National Cardiac PET Center, The Heart Failure Program, and the Cardiac Research Methods Center), Department of Medicine, University of Ottawa Heart Institute, Ottawa, ON, Canada; 3 University of Ottawa, Ottawa, ON, Canada; 4 Department of Radiology, The Ottawa Hospital, Ottawa, ON, Canada; 5 London Health Sciences Center, London, ON, Canada; 6 Montreal Heart Institute, Departments of Medicine and Radiology, Université de Montréal, Montreal, QC, Canada; 7 Dalhousie University, Halifax, NS, Canada; 8 Sunnybrook Health Sciences Center, Toronto, ON, Canada; 9 Helsinki University Central Hospital, Helsinki, Finland; 10 Université de Québec, Québec City, QC, Canada; 11 University of Calgary, Calgary, AB, Canada; 12 Turku PET Center, Turku, Finland; 13 Université de Sherbrooke, Sherbrooke, QC, Canada; 14 St. Michael’s Hospital, Toronto, ON, Canada; 15 Heart Center, Kuopio University Hospital, Kuopio, Finland

**Keywords:** Heart failure, Echocardiography, Cardiac magnetic resonance, Randomized controlled trial

## Abstract

**Background:**

Imaging has become a routine part of heart failure (HF) investigation. Echocardiography is a first-line test in HF given its availability and it provides valuable diagnostic and prognostic information. Cardiac magnetic resonance (CMR) is an emerging clinical tool in the management of patients with non-ischemic heart failure. Current ACC/AHA/CCS/ESC guidelines advocate its role in the detection of a variety of cardiomyopathies but there is a paucity of high quality evidence to support these recommendations.

The primary objective of this study is to compare the diagnostic yield of routine cardiac magnetic resonance versus standard care (that is, echocardiography with only selective use of CMR) in patients with non-ischemic heart failure. The primary hypothesisis that the routine use of CMR will lead to a more specific diagnostic characterization of the underlying etiology of non-ischemic heart failure. This will lead to a reduction in the non-specific diagnoses of idiopathic dilated cardiomyopathy and HF with preserved ejection fraction.

**Design:**

Tertiary care sites in Canada and Finland, with dedicated HF and CMR programs, will randomize consecutive patients with new or deteriorating HF to routine CMR or selective CMR. All patients will undergo a standard clinical echocardiogram and the interpreter will assign the most likely HF etiology. Those undergoing CMR will also have a standard examination and will be assigned a HF etiology based upon the findings. The treating physician’s impression about non-ischemic HF etiology will be collected following all baseline testing (including echo ± CMR). Patients will be followed annually for 4 years to ascertain clinical outcomes, quality of life and cost. The expected outcome is that the routine CMR arm will have a significantly higher rate of infiltrative, inflammatory, hypertrophic, ischemic and ‘other’ cardiomyopathy than the selective CMR group.

**Discussion:**

This study will be the first multicenter randomized, controlled trial evaluating the role of CMR in non-ischemic HF. Non-ischemic HF patients will be randomized to routine CMR in order to determine whether there are any gains over management strategies employing selective CMR utilization. The insight gained from this study should improve appropriate CMR use in HF.

**Trial registration:**

NCT01281384.

## Background

Innovations in imaging create the potential for earlier, less invasive and more accurate diagnoses of disease and may ultimately improve health outcomes. However, imaging is also one of the fastest growing health care expenditures. The 2005, the US Medicare Payment Advisory Committee found that the growth of cardiac imaging was nearly double any other cardiac procedure [1]. This underscores the need to ensure efficient development, implementation and use of this health care resource in order to provide value to patients and society. However an evaluation of the clinical and economic impact of cardiac imaging has lagged behind the pace of the technology. A recent review of current ACC/AHA guidelines found that only 11% of recommendations provide level of evidence A (multiple randomized controlled trials and meta analyses) and 48% have level of evidence C (consensus opinion) [2]. In cardiac diagnostics, level of evidence A is found in only 2%, level of evidence C in 17% and in 44% of recommendations there is no evidence provided. These observations underscore the need for more evidence based trials, particularly in cardiac imaging.

In 2007, the Canadian Institutes of Health Research New Frontiers Program consensus conference identified the urgent need for improved evaluation of imaging in relation to outcomes and translation from bench to bedside, particularly in heart failure (HF). In response, we established IMAGE-HF, a synergistic multi-disciplinary, multi-modality, multi-center Canada-Finland team comprised of seven Canadian sites and three Finnish sites. The main goal of IMAGE-HF is to evaluate cardiac imaging using existing clinical practice strategies for HF and their links to relevant outcomes (level 1 projects) and to create a unique translational platform for the evaluation of novel imaging biomarkers in animal models of HF and in HF patient populations (level 2 and 3 projects). IMAGE-HF 1-B, rOUTine versus Selective MAgnetic Resonance in non-ischemic HearT Failure-HF (OUTSMART-HF), will study the role of cardiac magnetic resonance (CMR) in non-ischemic heart failure.

### Statement of the problem

Population studies reveal that 50% of HF patients have preserved ejection fraction (HFPEF) and a dismal prognosis similar to those HF patients with reduced ejection fraction (HFREF) [3-5]. Among the HFREF patients, 50% have no significant underlying coronary artery disease [6]. The most common identifiable causes for non-ischemic HF include hypertension, valvular heart disease, arrhythmia, post-viral and acquired or inherited cardiomyopathies. However, standard measures of cardiac morphology and function obtained on imaging testing provide only limited information about HF etiology or prognosis [7] and, other than ejection fraction (EF), have a limited role in therapeutic decision-making.

Echocardiography is well suited for the assessment of HF patients because it is widely available and provides reliable information on cardiac performance in normal and diseased states. It is also considered the best imaging modality for valvular disease, a significant finding in 35% of patients hospitalized with HF [8]. More recent technological advances such as tissue velocity and strain have shed new insights into pathophysiological mechanisms that may be particularly pertinent in HFPEF [9-11]. Furthermore, diastolic dysfunction determined by Doppler echocardiography is common in HF patients and has been shown to be a predictor of increased mortality during longitudinal follow-up [12]. Data from the Rochester Epidemiologic Program revealed that there was a lower risk of death in HF patients who had undergone echocardiograms compared to those who had not, suggesting that echocardiography improved care and should be part of the routine investigation of HF patients [13]. Given the available evidence and its extensive clinical experience, echocardiography is recommended as a class 1 indication in the initial investigation of all HF patients [14-16].

CMR is emerging as a valuable tool in the diagnosis of non-ischemic heart failure and is considered preferable or at least equivalent to other diagnostic tests in several cardiomyopathies [17]. Ventricular volumes and function derived from CMR is more accurate than standard echocardiography due to high inter-test and inter-observer reproducibility [18]. Moreover, CMR offers insight into myocardial tissue characteristics [19], with specific regional patterns of myocardial injury [20]. Non-infarct and infarct patterns have been reported in 28% and 13%, respectively, of patients with HF and unobstructed coronary arteries [19]. In a recent single site study of 120 patients with new onset HF, CMR had a 97% accuracy for differentiating an ischemic from a non-ischemic etiology [21]. CMR has been used to characterize myocarditis [22-24], sarcoidosis [25], hypertrophic cardiomyopathy [26], hemochromatosis [27] and amyloidosis [28]. CMR thus offers a new class of diagnostic and prognostic parameters for personalized therapy and prevention of HF. However, there is a need to understand how such data impacts diagnosis, clinical decision-making and patient outcomes.

The diagnosis of HFPEF remains challenging as discussed in negative drug trials to date [29-31]. Diagnostic criteria incorporating clinical signs and symptoms, biomarkers, imaging and/or hemodynamic parameters have been proposed but not studied well [32]. Diffuse myocardial fibrosis as assessed by CMR has been utilized as a novel diagnostic target in HF [33] and cardiomyopathy subjects [34]. Patients with HFPEF are known to have increased myocardial collagen and fibrosis and hence CMR could be used to study this group.

Despite growing evidence of the utility for CMR in various cardiomyopathies, a comprehensive study examining its role in HF is lacking.

### Objectives

#### Primary objective

To compare the diagnostic yield of routine cardiac magnetic resonance versus standard care (that is, echocardiography with only selective use of CMR) in patients with non-ischemic heart failure. The diagnostic categories of HF to be considered in this study include: idiopathic dilated cardiomyopathy, infiltrative cardiomyopathy, inflammatory cardiomyopathy, hypertrophic cardiomyopathy, heart failure with preserved ejection fraction (HFPEF), ischemic cardiomyopathy, mixed etiology and ‘other’ (for example, pericardial disease, adult congenital heart disease, arrhythmogenic right ventricular cardiomyopathy).

#### Secondary objectives

To determine the effects that routine use of CMR in non-ischemic HF has on therapeutic decisions on the Composite Clinical Endpoint (CCE), cardiac function, symptoms, quality of life (QoL), and costs. Ancillary measurements will include the safety of imaging tests and adverse reactions to gadolinium contrast agent.

### Hypotheses

#### Primary hypothesis

Routine use of CMR (versus selective use) will lead to a more specific diagnostic characterization of the underlying etiology of non-ischemic heart failure. This will lead to a reduction in the non-specific diagnoses of idiopathic dilated cardiomyopathy and HFPEF.

#### Secondary hypotheses

Routine use of CMR will have a significant impact on treatment decisions, and will (1) lead to more disease specific therapies and/or (2) cause a significant change in the number and class of HF medications, during follow-up. The routine CMR group will also have improved clinical outcomes (CCE), symptoms and QoL and decreased costs compared to the standard of care group during follow-up.

### Design

**Figure 1:**
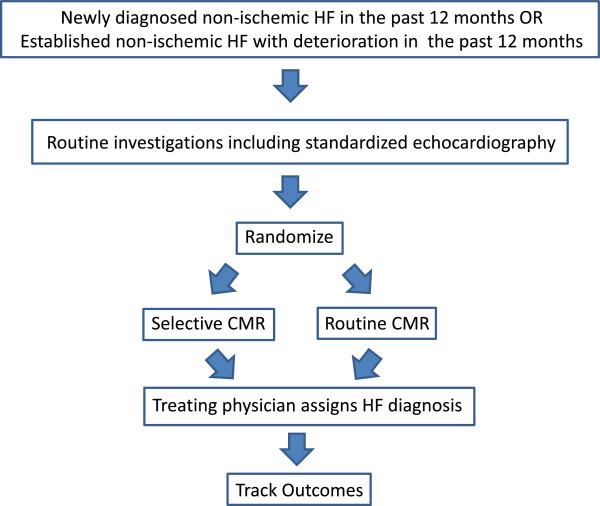
**Flow diagram for OUTSMART-HF.** Abbreviations: HF = heart failure and CMR = cardiac magnetic resonance.

Randomized controlled trial comparing i) routine CMR to ii) echocardiography with selective CMR in patient with HF due to a non-ischemic cardiomyopathy (NICM) and/or HFPEF. Among patients enrolled in level 1 of IMAGE-HF, it is expected that 504 will have known NICM (or strongly suspected based on young age, absent risk factors and presenting history) and/or HFPEF (Figure 1).

Tertiary care sites (in Canada and Finland) with dedicated HF programs will participate in the study (Additional file 1). Consecutive patients will be enrolled at sites with CMR programs (defined as a minimum 200 cases/year) and randomized to routine CMR or selective CMR. Non-ischemic HF patients from sites with smaller CMR programs will be included in a registry of patients undergoing routine HF care (that is, selective use of CMR). All sites will receive approval from local ethics committees (UOHI REB protocol # 2010422-01H) and patients will give written informed consent. Participants in the selective CMR arm may only undergo CMR when there is a suspicion or uncertainty for a diagnosis of: 1) infiltrative cardiomyopathy, 2) arrhythmogenic right ventricular cardiomyopathy, 3) adult congenital heart disease or 4) pericardial disease following standard HF care including echocardiography [35]. Other tertiary sites may be added in year two to three depending on recruitment needs and registry sites may become randomization sites if the experience and wait-time criteria are met.

### Study population

We will recruit patients with either newly diagnosed HF within the past 12 months or established HF patients with deterioration/decompensation within the past 12 months.

*Inclusion criteria:*

Patients with new or worsening HF as above *and*:

1) age > 18 years

2) working clinical diagnosis (known or highly suspected) of non-ischemic cardiomyopathy (NICM) or clinical diagnosis of HFPEF (signs or symptoms of heart failure with a LVEF ≥ 40%)

3) documented history of Class II-IV NYHA HF symptoms within the past 12 months

*Exclusion criteria:*

1) prior CMR and no major change in clinical condition

2) well-documented specific etiology (for example, known amyloidosis or hemochromatosis)

3) physician considers cause of heart failure is attributable to obstructive CAD

4) documented previous ST-segment elevation myocardial infarction (STEMI) (any territory) or non-ST-segment elevation myocardial infarction (NSTEMI) in the left anterior descending coronary artery territory

5) evidence of multi-vessel ischemia on stress imaging

6) ongoing need for advanced heart failure support (for example, inotropes or intra-aortic balloon pump)

7) severe valvular heart disease requiring surgery within the next six months

8) contraindications to CMR (for example, certain metallic implants, severe claustrophobia)

9) contraindications to gadolinium contrast agent (glomerular filtration rate < 30 ml/min/1.72 m^2^, pregnancy)

10) severe medical conditions that significantly affect the patient's outcome (for example, active malignancy)

11) inability to give informed consent

### Description of interventions (randomized and registry participants)

Baseline: demographic and clinical data will be collected from all participants on standardized case report forms. This data will be collected from the participant and the most recent, routine history and physical examination that have been completed by the treating physician. Quality of life questionnaires (Minnesota Living with Heart Failure (MLHFQ) and EuroQoL Quality of Life Scale (EQ5D)) will be administered. Patients at all sites will undergo standard HF investigations according to published Canadian guidelines [14,36,37]. Each center will have a HF lead, an echo lead and a CMR lead as well as a study co-ordinator.

#### Echocardiography

The performance and interpretation of echocardiography will be carried out according to established Canadian guidelines [38]. The echocardiography study and report must include the following measures: left ventricular (LV) size and systolic function (modified Simpson’s biplane formula), LV diastolic function (E/e’), LV mass (Teichholz formula), left atrial (LA) volume (biplane formula), valvular function (Doppler), right ventricular function, pulmonary arterial pressures (if possible) and pericardial assessment. Appendix A. The feasibility of implementing these standards and collaboration among multiple centers has been demonstrated in prospective echocardiography trials such as the ASTRONOMER study [39]. The new components such as strain and tissue velocity will be obtained and measured as described [40]. A standardized case report form will be completed by the echocardiographer. The most likely HF etiology based upon the echocardiogram will be provided to the referring physician.

#### CMR

Following all HF investigations (including echocardiography), study participants randomized to routine CMR as well as those requiring selective CMR will undergo a conventional scan. The CMR protocol must include an assessment of biventricular volumes and systolic function, LV mass, LA volume (biplane formula), global and regional myocardial edema using T2 weighted sequences, and hyperemia and regional myocardial fibrosis using contrast enhanced T1 weighted sequences. Appendix A. CMR interpreters participating in this trial should report a minimum of 100 cases/year. Local CMR interpreters at each participating center will be expected to complete a standardized clinical report form. The most likely HF etiology based upon the CMR will be provided to the referring physician. All CMR data acquisition and reporting will conform to current published guidelines [41,42].

The echo and CMR reports will be integrated with the other clinical data when used by the treating physician to make a final clinical determination on the HF etiology.

### Primary outcome measure - frequency of definitive diagnoses

Following the completion of all baseline testing (including echo ± CMR), the treating physician will assign a diagnosis on a standardized template using all available information. The HF categories include: idiopathic dilated cardiomyopathy, infiltrative cardiomyopathy, inflammatory cardiomyopathy, hypertrophic cardiomyopathy, heart failure with preserved ejection fraction (HFPEF), ischemic cardiomyopathy, mixed etiology and other (for example, pericardial disease, adult congenital heart disease, arrhythmogenic right ventricular cardiomyopathy). The diagnosis of non-ischemic cardiomyopathies will be based upon the 2008 Canadian Cardiovascular Society Consensus conference guidelines [37].

#### Expected result

The routine CMR arm will have a significantly higher rate of infiltrative, inflammatory, hypertrophic, ischemic, mixed and other cardiomyopathies than the selective CMR group.

### Follow-up

Clinical outcomes will be collected by telephone follow-up, and/or by hospital chart and electronic health record review at three and twelve months and then yearly up to four years, following completion of baseline scans. Data will be recorded on standardized case report forms. Quality of life questionnaires (MLHFQ and EQ5D) will be administered at each follow-up.

### Secondary outcome measures

(i) Treatment effects: at three months and one year, the treating physician will be asked to reassess the HF etiology. The presence of each HF medication class will be re-assessed in addition to the overall number of cardiac medications. The presence of advanced HF therapies will additionally be recorded at each follow-up visit including: implantable device, electrophysiologic study/ablation, cardiac surgery/transplantation, and disease specific therapies (for example, phlebotomy for hemochromatosis; steroids for sarcoidosis).

(ii) Clinical endpoints: Composite Clinical Endpoints (CCE that is, death, cardiovascular (CV) death, HF admission), left ventricular function, QoL, referral to HF clinic, costs and safety will be assessed at three months, then annually for a minimum of one year and a maximum of four years.

Resource utilization and costs: regression methods will be used to assess the incremental costs associated with the routine use of CMR.

(iii) Echo/CMR variability: anonymized copies of CMR and echo studies will be sent to a core lab for interpretation in approximately 10% of cases in order to assess reproducibility and quality assurance of the results. Appendix A.

### Statistical analysis

Descriptive statistics will be used to summarize the characteristics of the patients for each imaging technology on demographic, clinical and site related factors, and differences between these groups will be reviewed for their clinical significance.

#### Analysis populations

For the purposes of data analysis, three study populations will be considered: Intent-to-treat (ITT) population, As-treated population and Per-protocol population. The ITT population will be used for the main analysis for all primary and secondary objectives, except for the safety analysis where the As-treated population will be used. As a secondary analysis, the analyses will be repeated for the As-treated and Per-protocol populations.

#### Primary analysis

For the primary analysis, the definitive diagnosis of cardiomyopathy between routine CMR versus echo with selective CMR, diagnosis will be compared using chi-square techniques. Potential confounding variables of the relationship between the imaging strategies and the primary endpoint will be assessed. In particular, propensity scores based on patient factors (for example, in/outpatient, NYHA class, HF duration, diabetes, atrial fibrillation) and site factors (for example, time-to-imaging, time-to-therapy) will be used in the analysis if necessary to adjust for potential differences. A logistic regression model will be used to assess the occurrence of the endpoints between the imaging strategies (model will include a group indicator variable) adjusting for any pertinent baseline differences identified.

#### Secondary outcomes

For the binary secondary outcomes, such as CCE, referral to HF clinic and HF therapies, chi-square tests will be used to compare the imaging strategies. Logistic regression analysis will be used for adjusting any pertinent baseline differences identified. For continuous secondary outcomes, such as LV function, MLHFQ and EQ5D, analysis of variance will be used to compare trends over time between the imaging strategies. Analysis of covariance will be used for adjusting any pertinent baseline differences identified. For assessing the reproducibility of echo/CMR results, reliability statistics, including intra-class correlations and kappas, will be calculated to compare CMR and echo studies with the core lab interpretations.

#### Economic evaluation

A cost minimization analysis comparing routine CMR versus standard care (echocardiography plus selective CMR) will be conducted. Analysis will be restricted to the follow-up period within the study database and will be conducted from the health care system perspective. Regression analysis will provide 95% confidence intervals around the estimate of incremental costs. In addition, univariate sensitivity analysis will be conducted to assess the robustness of the study’s results to changing assumptions related to the unit costs of specific resource items.

#### Safety analysis

Safety will be evaluated by documenting all adverse events. Descriptive statistics (frequency distributions, numerical descriptors) and 95% confidence intervals will be calculated. The As-treated population will be the main analysis population for this safety evaluation.

#### Missing data

‘Missingness’ is considered to be missing at random and mixed methods repeated measures (MMRM) and multiple imputation techniques will be used for handling missing data. In particular, for continuous outcomes at multiple time points MMRM will be used.

#### Sample size justification

Based on a study of patients with NICM (prior to availability of CMR) a myocardial biopsy adds a specific diagnosis to standard approaches in 15% of patients with NICM increasing the rate from 35% to 50% [7]. For the sample size determination, the estimated occurrence of a definitive diagnosis with CMR is 50% and echo (with selective use of CMR) is 35%. For the primary analysis comparing routine CMR versus selective CMR with a sample size of 252 per group is required to detect a difference in the event rate of definitive diagnosis of cardiomyopathy of 35% (selective CMR) versus 50% (routine CMR), based on the Z-test with a pooled variance estimate, and after adjustment for 10% patient loss or withdrawal, we will be able to detect a difference in the event rate of definitive diagnosis of cardiomyopathy of 35% (selective CMR) versus 50% (routine CMR), a level of significance of 0.05, power of 90% and an adjustment for 10% patient loss or withdrawal.

### Study management

The IMAGE-HF trial is managed by an Executive Committee consisting of clinicians specialized in diagnostic imaging and/or heart failure and experts in biostatistics, physics and radiochemistry, as well as a larger Steering Committee consisting of members of the Executive Committee and representatives of all the initial study centers. In addition there is an events adjudication committee which will independently review and adjudicate each clinical event blinded to treatment randomization. Since all the imaging approaches are part of standard clinical practice, no interim analysis is planned but there will be an independent Data Safety Monitoring Board (DSMB) which will review the safety data on a periodic basis; the frequency of the meetings and the charter governing the DSMB will be finalized at the first meeting of the DSMB.

## Discussion

Multicenter randomized controlled trials are needed to evaluate the emerging role of advanced cardiac imaging in heart failure. CMR is increasingly advocated as a useful tool to evaluate the diagnosis and prognosis of non-ischemic HF patients, but to date there is little evidence to show that outcomes have improved. In IMAGE-HF 1-B, Canadian and Finnish tertiary care sites with access to CMR will randomize non-ischemic HF patients to routine CMR in order to determine whether there are any gains over current imaging strategies employing selective CMR utilization. All patients in this trial will undergo a comprehensive baseline echocardiogram as recommended in HF guidelines.

The design of this trial has several appealing aspects:

First, we will evaluate the role of CMR in HF in a real world setting. Given differences in MRI access, local imaging expertise and in the interpretation of guidelines, the use of CMR in non-ischemic HF can vary greatly in a tertiary care setting. In the proposed randomized trial, we will study whether routine CMR offer any diagnostic gains over a more judicious approach. Such a strategy will thus emulate centers with open CMR access versus those without and determine what if any impact this has on actual patient management. All imaging studies, echo and CMR, will be part of the overall clinical care received by the patient. We also feel that the multicenter design with varying level of CMR expertise and the inclusion of new or worsening HF patients will provide a real world experience.

Second, we will be able to determine whether CMR diagnoses in non-ischemic HF differ from echo diagnoses. The influence that the echo and CMR diagnoses have on the treating physician’s HF diagnosis will be examined. Also, the reproducibility of the imaging diagnoses will be determined in 10% of CMRs and echos as part of our quality assurance analysis.

Third, the potential for routine CMR to guide HF pharmacotherapy and to initiate disease specific treatment will be evaluated. CMR should uncover HF etiologies, such as myocardial infiltration, that was not previously detected by echo and other baseline testing. The treating physician could then prescribe treatment that is more tailored to the underlying pathology.

Fourth, we will determine the impact of routine CMR on the outcomes of non-ischemic HF patients. Most advanced cardiac imaging studies evaluate the diagnostic accuracy of a given modality with no assessment on patient outcome. In a secondary analysis, we will evaluate clinical outcomes including: a composite clinical end-point (death, CV death and HF admission) and two quality of life surveys: MLHFQ and EQ5D. We will also ascertain the feasibility of implementing a routine CMR approach in terms of safety and cost.

### Limitations

Given that CMR provides insight into myocardial characterization, we expect that the etiology of heart failure will be better characterized in those HF patients undergoing routine CMR. However, in some instances additional investigations such as myocardial biopsy and invasive hemodynamics may be required to confirm CMR findings. Therefore, the final diagnosis of heart failure etiology may ultimately be determined by CMR driven ancillary testing. We will assess the impact of downstream resource utilization on diagnosis and outcome in the multivariable analysis as well as calculate the additional cost burden.

### Summary

In largely single center studies, CMR has been shown to offer a diagnostic advantage in specific HF disease states however its role in undifferentiated non-ischemic HF is largely unknown. Thus IMAGE-HF is a clinically relevant and timely study in an era of growing health costs and increasing pressure for fiscal responsibility. Given the available evidence and local experiences, we are confident that routine CMR will offer diagnostic and therapeutic advantages that ultimately improve patient outcomes at affordable costs.

## Trial status

Recruiting

## Appendix A

Standardization and quality assurance (IMAGE-QA)

The IMAGE-HF QA program standardizes several important aspects of clinical imaging:

1. defining best current imaging practice for standard-care tests

2. disseminating advanced imaging technology and standards

3. promoting structured reporting and comprehensive imaging QA

4. ensuring consistent interpretation and patient management recommendations

For the IMAGE-HF project 1-B (OUTSMART-HF), this includes standard operating procedures (SOPs) for echocardiography (ECHO) and cardiac magnetic resonance (CMR), as well as structured reporting elements and quality assurance review by QA-CORE labs (SOPs and CRFs are posted on the IMAGE-HF website).

Standard-care imaging protocols

*Echocardiography (ECHO)*:

IMAGE-HF 1-AB SOP ECHO-etiology (MHI)

Based on Montreal Heart Institute (MHI) CORE laboratory procedure manual for the IMAGE-HF 1-A study (AIMI-HF): TECHNICAL GUIDE FOR ECHOCARDIOGRAPHY October 2010 (E O’Meara, J-C Tardif). Detailed procedures are described for the required parasternal, apical and sub-costal view measurements, and instructions for transfer of DICOM format images to the MHI CORE lab.

Advanced imaging protocols

*Cardiac Magnetic Resonance (CMR)*:

IMAGE-HF 1-B/2-A SOP CMR-etiology-fibrosis (CanSCMR)

Based on Canadian Society for Cardiovascular Magnetic Resonance (CanSCMR) protocol recommendations v1.3 April 2009. Imaging parameters are included for assessment of LV function, inflammation, fibrosis and mitral valve regurgitation.

Common structured reporting elements

*ECHO*:

HF18C 1-B-etiology ECHO Report

The following parameters are included on the interpretation CRF: LV structure, LV systolic and diastolic function parameters, LV segmental wall-motion scores, RV structure and systolic function, severity of valve stenosis and regurgitation. Clinical interpretation as selected from a list of the most likely echocardiographic diagnoses, and confirmation that the diagnosis was communicated to the referring physician, are captured on the CRF.

*CMR*:

HF19C 1-B-etiology CMR Report

The following parameters are included on the interpretation CRF: LV and RV structure and systolic function parameters, LV myocardial edema and hyperemia, LV segmental wall-motion and tissue characterization scores, severity of valve regurgitation, and assessment of pericardial effusion. Clinical interpretation as selected from a list of the most likely CMR diagnoses, and confirmation that the diagnosis was communicated to the referring physician, are captured on the CRF.

Quality assurance CORE lab reviews (QA-CORE)

A subset of scans (10%) are targeted for over-reading interpretation at an experienced site identified as the CORE lab for each imaging modality. The first two scans (and 5% of the subsequent scans) from each imaging modality at each recruiting site are transferred to the corresponding modality QA-CORE lab for clinical interpretation and comparison to the site interpretation for quality assurance. Disagreements in the overall ECHO or CMR diagnosis are resolved by subsequent consensus review between the site and CORE lab, and recorded on the corresponding CRFs: *HF18-QA, HF19-QA.*

The QA-CORE lab for ECHO is established at the University of Ottawa Heart Institute, and the QA-CORE lab for CMR is at the University of Alberta.

## Abbreviations

CCE: Composite clinical endpoint; CMR: Cardiac magnetic resonance; CV: Cardiovascular; DSMB: Data and safety monitoring board; echo: Echocardiography; EF: Ejection fraction; HF: Heart failure; HFPEF: Heart failure with preserved ejection fraction; HFREF: Heart failure with reduced ejection fraction; ITT: Intent-to-treat; LA: Left atrial; LV: Left ventricular; MHFQ: Minnesota living with heart failure questionnaire; MMRM: Mixed methods repeated measures; NICM: Non-ischemic cardiomyopathy; NSTEMI: Non-ST-segment elevation myocardial infarction; QoL: Quality of life; STEMI: ST-segment elevation myocardial infarction.

## Competing interests

MF: serves on the board of directors and a shareholder of Circle CV Imaging Inc, Calgary, AB, Canada, a cardiovascular MR imaging software manufacturer, E O’M: research funding from Johnson & Johnson for the cardiorenal-anemia syndrome in HF, BC: receives research and fellowship training support from GE Healthcare, research support from Pfizer and AstraZeneca, and educational support from TeraRecon Inc., R deK: consultant Jubilant DraxImage; research funding: Lantheus Medical Imaging, GE Healthcare and MDS Nordion; receives revenues from rubidium generator technology licensed to Jubilant DraxImage: receives revenues from FlowQuant software sales, RK: consultant Jubilant DraxImage; receives revenues from rubidium generator technology licensed to Jubilant DraxImage; receives revenues from FlowQuant software sales, GW: support from GE Healthcare for MR work which is helping to cover costs of the scans on the research system, RB: consultant Lantheus Medical Imaging, DraxImage; research funding: Lantheus Medical Imaging, GE, MDS Nordion. All other authors declare no competing interests.

## Authors’ contributions

IP conceived the study, participated in its design and coordination, provided CMR quality assurance and drafted the manuscript. KLC helped conceive the study, participated in its design and coordination and provided echocardiography quality assurance. RB helped conceive the study and participated in its design and oversaw overall project coordination. GAW helped conceive the study and participated in its design and coordination as well as providing statistical support. JK helped conceive the study and participated in its design and coordinated operations in the Finnish sites. LM helped conceive the study and participated in its design and coordination. EO helped conceive the study and participated in its design and coordination. BC helped conceive the study and participated in its design and coordination. LG is involved in the study design and project management and helped to draft the manuscript. RdK and RK established and will monitor the standardization of the imaging modalities. DC designed and will coordinate the economic evaluation. JE contributed to design of trial and will be involved with conducting the trial. JAW contributed to design of trial and will be involved with conducting the trial. MF contributed to design of trial and will be involved with conducting the trial. CD contributed to design of trial and will be involved with conducting the trial. AD contributed to design of trial and will be involved with conducting the trial. GD contributed to design of trial and will be involved with conducting the trial. MR contributed to design of trial and will be involved with conducting the trial. GW contributed to design of trial and will be involved with conducting the trial. ML contributed to design of trial and will be involved with conducting the trial. HH contributed to design of trial and will be involved with conducting the trial. EL contributed to design of trial and will be involved with conducting the trial. KC contributed to design of trial and will be involved with conducting the trial. HL-P contributed to design of trial and will be involved with conducting the trial. AH contributed to design of trial and will be involved with conducting the trial. RAD contributed to design of trial and will be involved with conducting the trial. LD contributed to design of trial and will be involved with conducting the trial. STY-H contributed to design of trial and will be involved with conducting the trial. AS contributed to design of trial and will be involved with conducting the trial. PF contributed to design of trial and will be involved with conducting the trial. JCT contributed to design of trial and will be involved with conducting the trial. MA contributed to design of trial and will be involved with conducting the trial. All authors read and approved the manuscript.

## Supplementary Material

Additional file 1: Table S1 IMAGE-HF Participating Sites. Click here for file

## References

[B1] Shaw LJ Narula J Cardiovascular imaging quality-more than a pretty picture! JACC Cardiovasc Imaging 2008 14 266 269 10.1016/j.jcmg.2008.01.005 19356437

[B2] Tricoci P Allen JM Kramer JM Califf RM Smith SC Jr Scientific evidence underlying the ACC/AHA clinical practice guidelines JAMA 2009 14 831 841 10.1001/jama.2009.205 19244190

[B3] Owan TE Hodge DO Herges RM Jacobsen SJ Roger VL Redfield MM Trends in prevalence and outcome of heart failure with preserved ejection fraction N Engl J Med 2006 14 251 259 10.1056/NEJMoa052256 16855265

[B4] Tribouilloy C Rusinaru D Mahjoub H Souliere V Levy F Peltier M Slama M Massy Z Prognosis of heart failure with preserved ejection fraction: a five year prospective population-based study Eur Heart J 2008 14 339 347 10.1093/eurheartj/ehm554 18156618

[B5] Bhatia RS Tu JV Lee DS Austin PC Fang J Haouzi A Gong Y Liu PP Outcome of heart failure with preserved ejection fraction in a population-based study N Engl J Med 2006 14 260 269 10.1056/NEJMoa051530 16855266

[B6] Horwich TB MacLellan WR Fonarow GC Statin therapy is associated with improved survival in ischemic and non-ischemic heart failure J Am Coll Cardiol 2004 14 642 648 10.1016/j.jacc.2003.07.049 14975476

[B7] Felker GM Thompson RE Hare JM Hruban RH Clemetson DE Howard DL Baughman KL Kasper EK Underlying causes and long-term survival in patients with initially unexplained cardiomyopathy N Engl J Med 2000 14 1077 1084 10.1056/NEJM200004133421502 10760308

[B8] Stein GY Kremer A Shochat T Bental T Korenfeld R Abramson E Ben-Gal T Sagie A Fuchs S The diversity of heart failure in a hospitalized population: the role of age J Card Fail 2012 14 645 653 10.1016/j.cardfail.2012.05.007 22858081

[B9] Nagueh SF Middleton KJ Kopelen HA Zoghbi WA Quinones MA Doppler tissue imaging: a noninvasive technique for evaluation of left ventricular relaxation and estimation of filling pressures J Am Coll Cardiol 1997 14 1527 1533 10.1016/S0735-1097(97)00344-6 9362412

[B10] Dokainish H Sengupta R Pillai M Bobek J Lakkis N Assessment of left ventricular systolic function using echocardiography in patients with preserved ejection fraction and elevated diastolic pressures Am J Cardiol 2008 14 1766 1771 10.1016/j.amjcard.2008.02.070 18549856

[B11] Yip GW Zhang Q Xie JM Liang YJ Liu YM Yan B Lam YY Yu CM Resting global and regional left ventricular contractility in patients with heart failure and normal ejection fraction: insights from speckle-tracking echocardiography Heart 2011 14 287 294 10.1136/hrt.2010.205815 21193686

[B12] Bursi F Weston SA Redfield MM Jacobsen SJ Pakhomov S Nkomo VT Meverden RA Roger VL Systolic and diastolic heart failure in the community JAMA 2006 14 2209 2216 10.1001/jama.296.18.2209 17090767

[B13] Senni M Rodeheffer RJ Tribouilloy CM Evans JM Jacobsen SJ Bailey KR Redfield MM Use of echocardiography in the management of congestive heart failure in the community J Am Coll Cardiol 1999 14 164 170 10.1016/S0735-1097(98)00523-3 9935024

[B14] Arnold JM Liu P Demers C Dorian P Giannetti N Haddad H Heckman GA Howlett JG Ignaszewski A Johnstone DE Jong P McKelvie RS Moe GW Parker JD Rao V Ross HJ Sequeira EJ Svendsen AM Teo K Tsuyuki RT White M Canadian Cardiovascular Society consensus conference recommendations on heart failure 2006: diagnosis and management Can J Cardiol 2006 14 23 45 10.1016/S0828-282X(06)70237-9 16450016 PMC2538984

[B15] Lindenfeld J Albert NM Boehmer JP Collins SP Ezekowitz JA Givertz MM Katz SD Klapholz M Moser DK Rogers JG Starling RC Stevenson WG Tang WH Teerlink JR Walsh MN HFSA 2010 comprehensive heart failure practice guideline J Card Fail 2010 14 e1 e194 10.1016/j.cardfail.2010.04.004 20610207

[B16] McMurray JJ Adamopoulos S Anker SD Auricchio A Bohm M Dickstein K Falk V Filippatos G Fonseca C Gomez-Sanchez MA Jaarsma T Kober L Lip GY Maggioni AP Parkhomenko A Pieske BM Popescu BA Ronnevik PK Rutten FH Schwitter J Seferovic P Stepinska J Trindade PT Voors AA Zannad F Zeiher A ESC guidelines for the diagnosis and treatment of acute and chronic heart failure 2012: the task force for the diagnosis and treatment of acute and chronic heart failure 2012 of the European society of cardiology. Developed in collaboration with the heart failure association (HFA) of the ESC Eur Heart J 2012 14 1787 1847 22611136 10.1093/eurheartj/ehs104

[B17] Kramer CM Budoff MJ Fayad ZA Ferrari VA Goldman C Lesser JR Martin ET Rajagopalan S Reilly JP Rodgers GP Wechsler L Creager MA Holmes DR Jr Merli G Newby LK Pina I Rodgers GP Weitz HH ACCF/AHA 2007 clinical competence statement on vascular imaging with computed tomography and magnetic resonance. A report of the American College of Cardiology Foundation/American Heart Association/American College of Physicians Task Force on Clinical Competence and Training J Am Coll Cardiol 2007 14 1097 1114 10.1016/j.jacc.2007.07.006 17825724

[B18] Strohm O Schulz-Menger J Pilz B Osterziel KJ Dietz R Friedrich MG Measurement of left ventricular dimensions and function in patients with dilated cardiomyopathy J Magn Reson Imaging 2001 14 367 371 10.1002/jmri.1052 11241808

[B19] McCrohon JA Moon JC Prasad SK McKenna WJ Lorenz CH Coats AJ Pennell DJ Differentiation of heart failure related to dilated cardiomyopathy and coronary artery disease using gadolinium-enhanced cardiovascular magnetic resonance Circulation 2003 14 54 59 10.1161/01.CIR.0000078641.19365.4C 12821550

[B20] Friedrich MG Abdel-Aty H Taylor A Schulz-Menger J Messroghli D Dietz R The salvaged area at risk in reperfused acute myocardial infarction as visualized by cardiovascular magnetic resonance J Am Coll Cardiol 2008 14 1581 1587 10.1016/j.jacc.2008.01.019 18420102

[B21] Assomull RG Shakespeare C Kalra PR Lloyd G Gulati A Strange J Bradlow WM Lyne J Keegan J Poole-Wilson P Cowie MR Pennell DJ Prasad SK Role of cardiovascular magnetic resonance as a gatekeeper to invasive coronary angiography in patients presenting with heart failure of unknown etiology Circulation 2011 14 1351 1360 10.1161/CIRCULATIONAHA.110.011346 21900085

[B22] Abdel-Aty H Boye P Zagrosek A Wassmuth R Kumar A Messroghli D Bock P Dietz R Friedrich MG Schulz-Menger J Diagnostic performance of cardiovascular magnetic resonance in patients with suspected acute myocarditis: comparison of different approaches J Am Coll Cardiol 2005 14 1815 1822 10.1016/j.jacc.2004.11.069 15936612

[B23] Mahrholdt H Goedecke C Wagner A Meinhardt G Athanasiadis A Vogelsberg H Fritz P Klingel K Kandolf R Sechtem U Cardiovascular magnetic resonance assessment of human myocarditis: a comparison to histology and molecular pathology Circulation 2004 14 1250 1258 10.1161/01.CIR.0000118493.13323.81 14993139

[B24] Friedrich MG Sechtem U Schulz-Menger J Holmvang G Alakija P Cooper LT White JA Abdel-Aty H Gutberlet M Prasad S Aletras A Laissy JP Paterson I Filipchuk NG Kumar A Pauschinger M Liu P Cardiovascular magnetic resonance in myocarditis: a JACC White Paper J Am Coll Cardiol 2009 14 1475 1487 10.1016/j.jacc.2009.02.007 19389557 PMC2743893

[B25] Smedema JP Snoep G van Kroonenburgh MP van Geuns RJ Dassen WR Gorgels AP Crijns HJ Evaluation of the accuracy of gadolinium-enhanced cardiovascular magnetic resonance in the diagnosis of cardiac sarcoidosis J Am Coll Cardiol 2005 14 1683 1690 10.1016/j.jacc.2005.01.047 15893188

[B26] Choudhury L Mahrholdt H Wagner A Choi KM Elliott MD Klocke FJ Bonow RO Judd RM Kim RJ Myocardial scarring in asymptomatic or mildly symptomatic patients with hypertrophic cardiomyopathy J Am Coll Cardiol 2002 14 2156 2164 10.1016/S0735-1097(02)02602-5 12505229

[B27] Anderson LJ Holden S Davis B Prescott E Charrier CC Bunce NH Firmin DN Wonke B Porter J Walker JM Pennell DJ Cardiovascular T2-star (T2*) magnetic resonance for the early diagnosis of myocardial iron overload Eur Heart J 2001 14 2171 2179 10.1053/euhj.2001.2822 11913479

[B28] Maceira AM Joshi J Prasad SK Moon JC Perugini E Harding I Sheppard MN Poole-Wilson PA Hawkins PN Pennell DJ Cardiovascular magnetic resonance in cardiac amyloidosis Circulation 2005 14 186 193 10.1161/01.CIR.0000152819.97857.9D 15630027

[B29] Yusuf S Pfeffer MA Swedberg K Granger CB Held P McMurray JJ Michelson EL Olofsson B Ostergren J Effects of candesartan in patients with chronic heart failure and preserved left-ventricular ejection fraction: the CHARM-Preserved Trial Lancet 2003 14 777 781 10.1016/S0140-6736(03)14285-7 13678871

[B30] Ahmed A Rich MW Fleg JL Zile MR Young JB Kitzman DW Love TE Aronow WS Adams KF Jr Gheorghiade M Effects of digoxin on morbidity and mortality in diastolic heart failure: the ancillary digitalis investigation group trial Circulation 2006 14 397 403 10.1161/CIRCULATIONAHA.106.628347 16864724 PMC2628473

[B31] Cleland JG Tendera M Adamus J Freemantle N Polonski L Taylor J The perindopril in elderly people with chronic heart failure (PEP-CHF) study Eur Heart J 2006 14 2338 2345 10.1093/eurheartj/ehl250 16963472

[B32] Paulus WJ Tschope C Sanderson JE Rusconi C Flachskampf FA Rademakers FE Marino P Smiseth OA De KG Leite-Moreira AF Borbely A Edes I Handoko ML Heymans S Pezzali N Pieske B Dickstein K Fraser AG Brutsaert DL How to diagnose diastolic heart failure: a consensus statement on the diagnosis of heart failure with normal left ventricular ejection fraction by the Heart Failure and Echocardiography Associations of the European Society of Cardiology Eur Heart J 2007 14 2539 2550 10.1093/eurheartj/ehm037 17428822

[B33] Iles L Pfluger H Phrommintikul A Cherayath J Aksit P Gupta SN Kaye DM Taylor AJ Evaluation of diffuse myocardial fibrosis in heart failure with cardiac magnetic resonance contrast-enhanced T1 mapping J Am Coll Cardiol 2008 14 1574 1580 10.1016/j.jacc.2008.06.049 19007595

[B34] Dass S Suttie JJ Piechnik SK Ferreira VM Holloway CJ Banerjee R Mahmod M Cochlin L Karamitsos TD Robson MD Watkins H Neubauer S Myocardial tissue characterization using magnetic resonance noncontrast t1 mapping in hypertrophic and dilated cardiomyopathy Circ Cardiovasc Imaging 2012 14 726 733 10.1161/CIRCIMAGING.112.976738 23071146

[B35] Hendel RC Patel MR Kramer CM Poon M Hendel RC Carr JC Gerstad NA Gillam LD Hodgson JM Kim RJ Kramer CM Lesser JR Martin ET Messer JV Redberg RF Rubin GD Rumsfeld JS Taylor AJ Weigold WG Woodard PK Brindis RG Hendel RC Douglas PS Peterson ED Wolk MJ Allen JM Patel MR ACCF/ACR/SCCT/SCMR/ASNC/NASCI/SCAI/SIR 2006 appropriateness criteria for cardiac computed tomography and cardiac magnetic resonance imaging: a report of the American College of Cardiology Foundation Quality Strategic Directions Committee Appropriateness Criteria Working Group, American College of Radiology, Society of Cardiovascular Computed Tomography, Society for Cardiovascular Magnetic Resonance, American Society of Nuclear Cardiology, North American Society for Cardiac Imaging, Society for Cardiovascular Angiography and Interventions, and Society of Interventional Radiology J Am Coll Cardiol 2006 14 1475 1497 10.1016/j.jacc.2006.07.003 17010819

[B36] Arnold JM Howlett JG Dorian P Ducharme A Giannetti N Haddad H Heckman GA Ignaszewski A Isaac D Jong P Liu P Mann E McKelvie RS Moe GW Parker JD Svendsen AM Tsuyuki RT O'Halloran K Ross HJ Rao V Sequeira EJ White M Canadian Cardiovascular Society Consensus Conference recommendations on heart failure update 2007: prevention, management during intercurrent illness or acute decompensation, and use of biomarkers Can J Cardiol 2007 14 21 45 10.1016/S0828-282X(07)70211-8 17245481 PMC2649170

[B37] Malcom J Arnold O Howlett JG Ducharme A Ezekowitz JA Gardner MJ Giannetti N Haddad H Heckman GA Isaac D Jong P Liu P Mann E McKelvie RS Moe GW Svendsen AM Tsuyuki RT O'Halloran K Ross HJ Sequeira EJ White M Canadian Cardiovascular Society Consensus Conference guidelines on heart failure - 2008 update: best practices for the transition of care of heart failure patients, and the recognition, investigation and treatment of cardiomyopathies Can J Cardiol 2008 14 21 40 10.1016/S0828-282X(08)70545-2 18209766 PMC2631246

[B38] Sanfilippo AJ Bewick D Chan KL Cujec B Dumesnil JG Honos G Munt B Sasson Z Tam J Tomlinson C Aboguddah A Ahmed S Ali M Arsenault M Ascah K Ashton T Baird M Basmadjian A Beique F Blakeley M Blais MJ Burggraf G Burwash I Cochrane J Fagan S Giannoccaro P Hughes W Jones A Jue J Koilpillai C Guidelines for the provision of echocardiography in Canada: recommendations of a joint Canadian Cardiovascular Society/Canadian Society of Echocardiography Consensus Panel Can J Cardiol 2005 14 763 780 16082436

[B39] Chan KL Teo K Dumesnil JG Ni A Tam J Effect of lipid lowering with rosuvastatin on progression of aortic stenosis: results of the aortic stenosis progression observation: measuring effects of rosuvastatin (ASTRONOMER) trial Circulation 2010 14 306 314 10.1161/CIRCULATIONAHA.109.900027 20048204

[B40] Gorcsan J III Tanaka H Echocardiographic assessment of myocardial strain J Am Coll Cardiol 2011 14 1401 1413 10.1016/j.jacc.2011.06.038 21939821

[B41] Hundley WG Bluemke D Bogaert JG Friedrich MG Higgins CB Lawson MA McConnell MV Raman SV van Rossum AC Flamm S Kramer CM Nagel E Neubauer S Society for Cardiovascular Magnetic Resonance guidelines for reporting cardiovascular magnetic resonance examinations J Cardiovasc Magn Reson 2009 14 5 10.1186/1532-429X-11-5 19257889 PMC2662831

[B42] Friedrich MG Larose E Patton D Dick A Merchant N Paterson I Canadian society for cardiovascular magnetic resonance (CanSCMR) recommendations for cardiovascular magnetic resonance image analysis and reporting Can J Cardiol 2013 14 260 265 10.1016/j.cjca.2012.07.007 23010085

