# Supplementary material for: Routine versus selective cardiac magnetic resonance in non-ischemic heart failure – OUTSMART-HF: study protocol for a randomized controlled trial (IMAGE-HF (heart failure) project 1-B)
Source: Trials. 2013 Oct 12;14:332. doi: 10.1186/1745-6215-14-332 (PMC4016591; doi:10.1186/1745-6215-14-332)
Supplement: Additional file 1: Table S1 — IMAGE-HF Participating Sites. [file 1745-6215-14-332-S1.doc]

Additional file

**Table S1.** IMAGE-HF Participating Sites

| IMAGE-HF Participating Sites |  |
| --- | --- |
| University of Ottawa Heart Institute |  |
| R Beanlands | Co-Principal Investigator IMAGE-HF, Canada* |
| GA Wells | Principal Investigator CRMC* |
| R deKemp | Principal Investigator QA Program* |
| D Birnie | Co-Principal Investigator Project IIA |
| L Mielniczuk | Co-Principal Investigator Project IA |
| K Chan | Site Principal Investigator |
| B Chow | Principal Investigator Project IC |
| L Garrard | Project Management |
| R Hessian | Investigator |
| T Ruddy | Investigator |
| RA Davies | Investigator |
| H Haddad | Investigator |
| A Dick | Investigator |
| C Dennie | Investigator |
| D Coyle | Investigator |
| B McArdle | Investigator |
| T Dowsley | Investigator |
| G Dwivedi | Investigator |
| J DaSilva | Investigator |
| C Kelly | Research Coordinator |
| E Moga | Research Coordinator |
| R Klein | Core Lab Manager |
| K Williams | Statistician |
| R Fleming | Research Coordinator |
| M Boomgaardt | Research Coordinator |
| Montreal Heart Institute-Université de Montréal |  |
| JC Tardif | Investigator* |
| E O'Meara | Co-Principal Investigator Project IA |
| M Friedrich | Investigator* |
| J Rouleau | Investigator |
| T Heinonen | Investigator |
| F Marcotte | Investigator |
| N Racine | Investigator |
| HQ Ly | Investigator |
| J Morrissette | Research Coordinator |
| H. Brown | Research Coordinator |
| University of Alberta |  |
| I Paterson | Principal Investigator Project IB |
| L Lalonde | Investigator |
| J Ezekowitz | Investigator |
| M Irwin | Research Coordinator |
| University of Turku |  |
| J Knuuti | Co-Principal Investigator IMAGE-HF, Finland* |
| H Ukkonen | Investigator |
| S Yla-Herttuala | Investigator* |
| H Leskinen | Investigator |
| A Saraste | Investigator |
| T Vasankari | Research Coordinator |
| K Lahtonen | Research Coordinator |
| M Tarkia | Site Project Manager |
| University Central Hospital, Helsinki |  |
| M Laine | Site Principal Investigator |
| H Hanninen | Investigator |
| M Pietila | Research Coordinator |
| Heart Center, Kuopio University Hospital |  |
| J Hartikainen | Site Principal Investigator |
| Marja Hedman | Investigator |
| S Karkkainen | Investigator |
| I Kaivonurmi | Research Coordinator |
| M Sutinen | Research Coordinator |
| Sunnybrook Health Sciences Center |  |
| G Wright | Site Co-Principal Investigator* |
| K Connelly | Site Co-Principal Investigator |
| R Myers | Investigator |
| C Cunningham | Investigator |
| E Crystal | Investigator |
| A Leber | Investigator |
| M Mohammed | Research Coordinator |
| J Malko | Research Coordinator |
| University of Calgary |  |
| A Howarth | Site Co-Principal Investigator |
| T Anderson | Site Co-Principal Investigator |
| A Krysk | Investigator |
| S Hutchison | Investigator |
| N Merchant | Investigator |
| S Weeks | Investigator |
| R Sandonato | Research Coordinator |
| S Rivest | Research Coordinator |
| J Veenhuyzen | Research Coordinator |
| M Seib | Research Coordinator |
| B Madden | Research Coordinator |
| D Durand | Research Coordinator |
| London Health Sciences |  |
| M Arnold | Site Principal Investigator* |
| G Wisenberg | Investigator |
| T Lee | Investigator |
| F Prato | Investigator |
| J White | Co-Principal Investigator Project IIA |
| K Carter | Research Coordinator |
| Laval University |  |
| E Larose | Site Principal Investigator |
| P Pibarot | Investigator* |
| B Cantin | Investigator |
| J Carange | Research Coordinator |
| K Bibeau | Research Coordinator |
| St. Michael's Hospital |  |
| M Freeman | Site Co-Principal Investigator |
| K Connelly | Site Co-Principal Investigator |
| H Leong-Poi | Investigator |
| G Moe | Investigator |
| A Al-Hesayen | Investigator |
| J Sloninko | Research Coordinator |
| Hamilton |  |
| V Tandon | Site Principal Investigator |
| K Gulenchyn | Investigator |
| F Spence | Investigator |
| A Khoorshed | Research Coordinator |
| Sherbrooke |  |
| E Turcotte | Site Principal Investigator |
| S Lepage | Investigator |
| Paul Farand | Investigator |
| S Joncas | Resident, recruitment |
| E Lavallee | Research Coordinator |
| Halifax |  |
| M Rajda | Site Principal Investigator |
| R Stewart | Investigator |
| J Clarke | Investigator |
| S Burrell | Investigator |
| B Clarke | Investigator |
| S Yarn | Research Coordinator |
| M MacFarlane | Research Coordinator |
| Winnipeg |  |
| M Kass | Site Principal Investigator |
| J Tam | Investigator |
| T Moore | Research Coordinator |
| A Munoz | Research Coordinator |
| QA Core Labs |  |
| R deKemp | Investigator - QA and Standardization Lead PET, SPECT QA Core Lab Team Leader (Ottawa) |
| R Klein | Core Lab Manager (Ottawa) |
| B McArdle | PET, SPECT QA Core Lab (Ottawa) |
| J Renaud | PET, SPECT QA Core Lab (Ottawa) |
| K Chan | ECHO QA Core Lab Team Leader (Ottawa) |
| J White | CMR QA Core Lab 1A Team Leader (London) |
| I Pauchard | CMR QA Core Lab 1A (London) |
| I Paterson | CMR QA Core Lab 1B Team Leader (Edmonton) |
| P L’Allier | ICA QA Core Lab Team Leader (Montreal) |
| B Chow | CTA QA Core Lab Team Leader (Ottawa) |
| Steering Committee* |  |
| R Beanlands |  |
| GA Wells |  |
| J Knuuti |  |
| M Friedrich |  |
| G Wright |  |
| M Arnold |  |
| JC Tardif |  |
| P Pibarot |  |
| S Ylä-Herttuala |  |
| R deKemp |  |
| DSMB |  |
| A Krahn, Chair |  |
| J Fallavollita |  |
| L Thabane |  |
| Events |  |
| H Haddad, Chair |  |
| DS Beanlands |  |
| L Duchesne |  |
| J Ezekowitz |  |
| R A Davies |  |
